# Carbon Dioxide and the Carbamate Post-Translational Modification

**DOI:** 10.3389/fmolb.2022.825706

**Published:** 2022-03-01

**Authors:** Lynsay I. Blake, Martin J. Cann

**Affiliations:** Department of Biosciences, Durham University, Durham, United Kingdom

**Keywords:** carbon dioxide, post-translational modification, carbamate, haemoglobin, rubisco, connexin, ubiquitin

## Abstract

Carbon dioxide is essential for life. It is at the beginning of every life process as a substrate of photosynthesis. It is at the end of every life process as the product of post-mortem decay. Therefore, it is not surprising that this gas regulates such diverse processes as cellular chemical reactions, transport, maintenance of the cellular environment, and behaviour. Carbon dioxide is a strategically important research target relevant to crop responses to environmental change, insect vector-borne disease and public health. However, we know little of carbon dioxide’s direct interactions with the cell. The carbamate post-translational modification, mediated by the nucleophilic attack by carbon dioxide on *N*-terminal α-amino groups or the lysine ɛ-amino groups, is one mechanism by which carbon dioxide might alter protein function to form part of a sensing and signalling mechanism. We detail known protein carbamates, including the history of their discovery. Further, we describe recent studies on new techniques to isolate this problematic post-translational modification.

## Introduction

Since its discovery in gas exhaled from the lung in 1757 (West, 2004, 2014) carbon dioxide (CO_2_) has been recognised as a critical component of biological processes throughout the biosphere. Its contribution to the essential physiological processes of metabolism, photosynthesis, chemosensing, and cellular homeostasis (Cummins et al., 2020) has meant that organisms across the three domains of life had evolved mechanisms to sense, transport, and respond to CO_2_ (Cummins et al., 2014). Although a great deal of knowledge exists about the physiological processes where CO_2_ is produced or consumed, less is known about the direct mechanisms of CO_2_ interactions with biomolecules.

**FIGURE 1:**
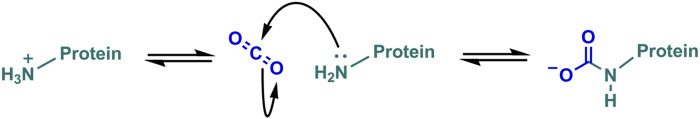
Carbamates form through the reversible reaction between CO_2_ and neutral amine groups.

One way in which CO_2_ has been shown to interact with protein directly is through carbamylation of neutral *N*-terminal -amino or lysine ɛ-amino groups (Figure 1). This carbamate post-translational modification (PTM) is critical to regulating oxygen-binding in haemoglobin and the activation of the CO_2_-fixing enzyme RuBisCO. It has been suggested that protein carbamylation could form the basis of a widespread mechanism for biological regulation (Morrow et al., 1974; Lorimer, 1983). Computational studies predict that carbamates may be found in more than 1.3% of large proteins (Jimenez-Morales et al., 2014).

In this review, we bring together contextual examples of carbamylation and explore recent computational and experimental approaches with the potential to uncover the distribution of protein carbamylation within proteomes.

### CO_2_ and Haemoglobin

The linked processes of ventilation and metabolism are essential to survival in higher animals. Following ventilation, gas transfer enables oxygen to be provided promptly to the cells and their mitochondria, which is used in the final process of aerobic cellular respiration (oxidative phosphorylation). Oxidative phosphorylation coordinates with the tricarboxylic acid (TCA) cycle to form adenosine triphosphate (ATP) and the metabolites required by the organism for survival, producing CO_2_ as a waste product (Martínez-Reyes and Chandel, 2020). CO_2_ exits the cells to the bloodstream and is transported to the lungs for excretion, thus contributing to pH homeostasis (Cummins et al., 2020).

The link between ventilation and metabolism was first made in 1777 by Antoine Lavoisier with the observation that “*Eminently respirable air [oxygen] that enters the lung, leaves it in the form of chalky aeroform acids [carbon dioxide]… in almost equal volume….*” (West, 2014). At this time, it was presumed that the process of metabolism (“*slow combustion*”) was performed within the lung (West, 2014). By the mid to late 1800s it was clear that O_2_ was transported *via* the blood to tissues (where metabolism occurred) by the formation of a loose, dissociable interaction with haemoglobin (oxyhaemoglobin) (Barcroft and Hill, 1910; Saha et al., 2014), and CO_2_ was returned to the lung by similar means (Severinghaus and Bradley, 1958; Giegé, 2013).

In 1904 Bohr, Hasselbalch and Krogh measured haemoglobin oxygenation in canine blood and described the sigmoidal (rather than hyperbolic) nature of the oxyhaemoglobin dissociation curve (Bohr et al., 1904). This experiment demonstrated that increasing pCO_2_ resulted in a lowered affinity of haemoglobin for O_2_ (known as the Bohr effect). Conversely, Christiensen et al. (1914) described that increasing pO_2_ resulted in a decreased affinity of haemoglobin for CO_2_ (known as the Haldane effect). The Bohr and Haldane effects were reversible, and observed in various mammalian systems (Christiansen et al., 1914; West, 2019). Combined, the Bohr-Haldane effect results in haemoglobin being an efficient O_2_ transporter from the lungs to tissues and CO_2_ from the tissues to the lungs (Eaton et al., 1999). The nature of the oxyhaemoglobin dissociation curve led to the hypothesis that multiple O_2_ binding sites on haemoglobin acted cooperatively (Barcroft and Hill, 1910; Hill, 1913).

Haemoglobins belongs to a large family of proteins with members distributed across all three domains of life. The first structures (myoglobin and equine haemoglobin) were determined by X-ray crystallography in the 1950s (Perutz et al., 1960, 1964). Human adult haemoglobin is a tetramer consisting of two α and two β subunits similar in structure and size. The α and β subunits are formed of seven and eight helixes, respectively (A–H), joined by non-helical segments. Each subunit binds a heme group consisting of a porphyrin ring that coordinate a Fe^2+^ ion (capable of binding to O_2_) by four nitrogen atoms at its centre. The oxygenated and deoxygenated haemoglobin quaternary structures differ. The gap between two polypeptide chains in the haemoglobin molecule narrows when O_2_ binds to the Fe^2+^ (Paoli et al., 1996; Park et al., 2006; Perutz et al., 1960, 1964). The binding of the first O_2_ to the haemoglobin subunit enhances the ability of subsequent O_2_ molecules to bind to the remaining subunits (Adair, 1925; Pauling, 1935). This knowledge was used to develop the Monod Wyman and Changeux “two-state concerted” model for allostery where deoxygenated haemoglobin exists in a tense (T) state (with relatively low O_2_ affinity). When Fe^2+^ binds O_2,_ there is a movement of Fe^2+^ into the heme plane, which triggers a transition to the relaxed (R) state. In this R state, the remaining binding sites are more exposed and have an increased O_2_ affinity (Monod and Jacob, 1961; Bringas et al., 2017). Additional allosteric sites on haemoglobin were available for binding allosteric modulators, including H^+^, CO_2_, 2,3-diphosphoglycerate (2,3-DPG) and Cl^−^ (Perutz, 1970; Perrella and Russo, 2003; Yuan et al., 2015).

In the 1930s, CO_2_ was shown to combine rapidly and reversibly with haemoglobin to form carbaminohaemoglobin (Ferguson and Roughton, 1934; Stadie, 1935; Stadie and O’Brien, 1936). It was suggested that carbaminohaemoglobin formation was possible at multiple amino sites on haemoglobin (Stadie and O’Brien, 1936). Confirmation of carbamate formation at the *N*-terminal of the valines of each of the four human deoxyhaemoglobin chains was performed using cyanate-based blocking, which inhibited the uptake of CO_2_ by haemoglobin (Kilmartin and Rossi-Bernardi, 1969) and confirmed that CO_2_-binding was O_2_-linked (at constant pCO_2_ and pH, deoxyhemoglobin forms more haemoglobin-CO_2_ than haemoglobin-O_2_). The CO_2_ binding site was demonstrated to occur at the Val-1β site and linked to the O_2_-binding state of the β-chain (Matthew et al., 1977). Perrella et al., 1975 found that the adduct formed on the β chain is more prominent than the α chain. Physiologically, the reaction between the α-amino group and CO_2_ stabilises the protein’s deoxygenated form. Morrow et al., 1974 suggested that carbamino formation may be a general and functionally important phenomenon throughout biology and not limited to haemoglobin (Morrow et al., 1974).

### CO_2_ and Ribulose 1,5-Bisphosphate Carboxylase-Oxygenase (RuBisCO)

Thought to have emerged about three billion years ago during the Archaean aeon and before the Great Oxygenation Events (Kacar et al., 2017; Banda et al., 2020), the RuBisCO family of proteins is one of the most abundant on the planet (Ellis, 1979). Initially discovered in the 1940s by Wildman and Bonner (Wildman, 2002), these enzymes are represented across the three domains of life and grouped as structurally distinct operational forms based on protein sequence and secondary and tertiary structure (Duff et al., 2000; Schneider et al., 1990, 1992). Forms I, II and III catalyse the carboxylation (and oxygenation) of ribulose 1,5-bisphosphate, while form IV contains RuBisCO-like proteins (RLP), which although sequentially and structurally similar, perform distinct biological functions (Watson et al., 1999; Tabita et al., 2008; Kono et al., 2017).

RuBisCO I is found in plants, cyanobacteria, algae, and some proteobacteria and is responsible for the vast majority of atmospheric CO_2_ fixation through the Calvin-Benson-Bassham (CBB) reductive pentose phosphate pathway (Andersson and Backlund, 2008; the dark reactions of photosynthesis). Most commonly, Form I is a hexadecamer of eight large and eight small subunits (L8S8). There are four Form I subtypes: A and B in cyanobacteria, eukaryotic algae, and higher plants; C and D are found in nongreen algae and phototropic bacteria (Stec, 2012). It has been demonstrated that although operational RuBisCO forms differ in overall structure, all share a similar catalytic subunit dimer (Duff et al., 2000; Schneider et al., 1990; Tabita et al., 2007, 2008), formed from the interaction of the large subunits (the *C*-terminus of the first interacting with the *N*-terminus of the second to form two active sites with residues from both). It is thought that RuBisCO remains in the open state when receiving substates and eliminating products. Still, when catalytic events occur, it is in a closed state, essentially sequestering the active site from the bulk solvent (Duff et al., 2000).

**FIGURE 2:**
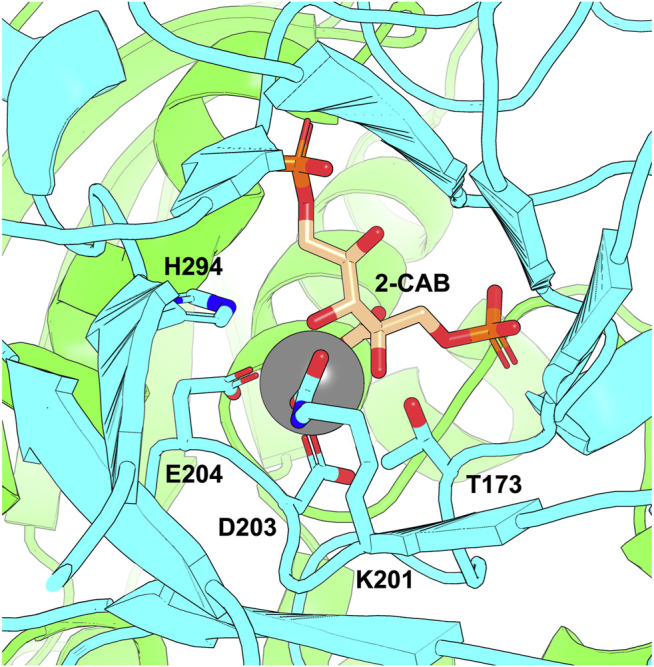
The RuBisCO active site showing the K201 carbamate. The figure shows the active site of *Arabidopsis thaliana* RuBisCO (PDB: 5IU0) with two large subunit chains shown in green and cyan and bound transition-state analogue 2-carboxy-D-arabinitol-1,5-bisphosphate (2-CAB). D203 and E204 coordinate Mg^2+^ co-factor (grey sphere) in the active site. The carbamate forms on K201 and is stabilised through interactions that include H294 and T173.

RuBisCO requires the catalytic site to be activated (Tabita et al., 2007, 2008) by sequential carbamylation of a specific lysine side chain followed by Mg^2+^ binding (Stec, 2012) (Figure 2). Once activated, RuBisCO’s role within the CBB is in catalysing the initial stage of carboxylation of ribulose 1,5-bisphosphate (RuBP) and cleavage to form two molecules of 3-phosphoglycerate (3-PGA) (one of which is used in RuBP regeneration, the other diverted to sustain biosynthesis of sugars and other high energy compounds). However, once activated, RuBisCO also catalyses a competitive oxygenation reaction, leading to the formation of one molecule of 3-PGA and one molecule of 2-phosphoglycolate (2-PG), which inhibits central carbon metabolism (Tcherkez, 2016). RuBisCO is a relatively inefficient enzyme with a low turnover rate (Bathellier et al., 2018). The turnover rate is further reduced by competition from the oxygenation reaction with the potential to reduce carbon fixation by up to 50% (Andersson and Taylor, 2003; Andersson, 2007). Therefore, optimisation of the carboxylation action of RuBisCO has been explored to improve crop efficiency and climate-resilient photosynthesis (Andersson and Backlund, 2008; Erb and Zarzycki, 2018; Valegård et al., 2018).

Identification of the RuBisCO catalytic site and its activation were, in most part, uncovered through kinetic and physical investigations in the 1960s and 70s. It became apparent that the sequential addition of CO_2_ (Lorimer et al., 1976) and Mg^2+^ (Miziorko and Mildvan, 1974; Lorimer et al., 1976) was required for activation. It was further demonstrated that this occurred through CO_2_.Mg^2+^.Enzyme complex formation (Miziorko and Mildvan, 1974). Further experiments demonstrated that the CO_2_ involved in the activation process was distinct from the substrate CO_2_ used in the subsequent carboxylase reaction (Miziorko, 1979; Lorimer and Miziorko, 1980) and that the catalytic site was located on the RuBisCO large subunit (Nishimura and Akazawa, 1974; Lorimer and Miziorko, 1980; Tabita et al., 2007). The hypothesis that activation was through carbamate formation on the ε-amino group of a lysine residue (Lorimer et al., 1976) was confirmed by Lorimer and Miziorko (1980), building on the work of (Ephraim Katchalski et al., 1951) and (Akoyunoglou et al., 1967). This finding was followed by identifying the carbamylation site on Lys-201 (Lorimer, 1981) close to the Mg^2+^ co-factor binding site (Pierce and Reddy, 1986). Based on the observed carbamylation of Haemoglobin and RuBisCO, George Lorimer proposed carbamate modification of *N-*terminal α-amino and lysine ε-amino groups as the basis of a widespread mechanism for CO_2_ detection. However, as observed by Professor Lorimer, there was no available method for trapping these carbamates on protein to enable their identification. The carbamate modification was, therefore, largely forgotten.

## Discovery of Other Carbamates

**FIGURE 3:**
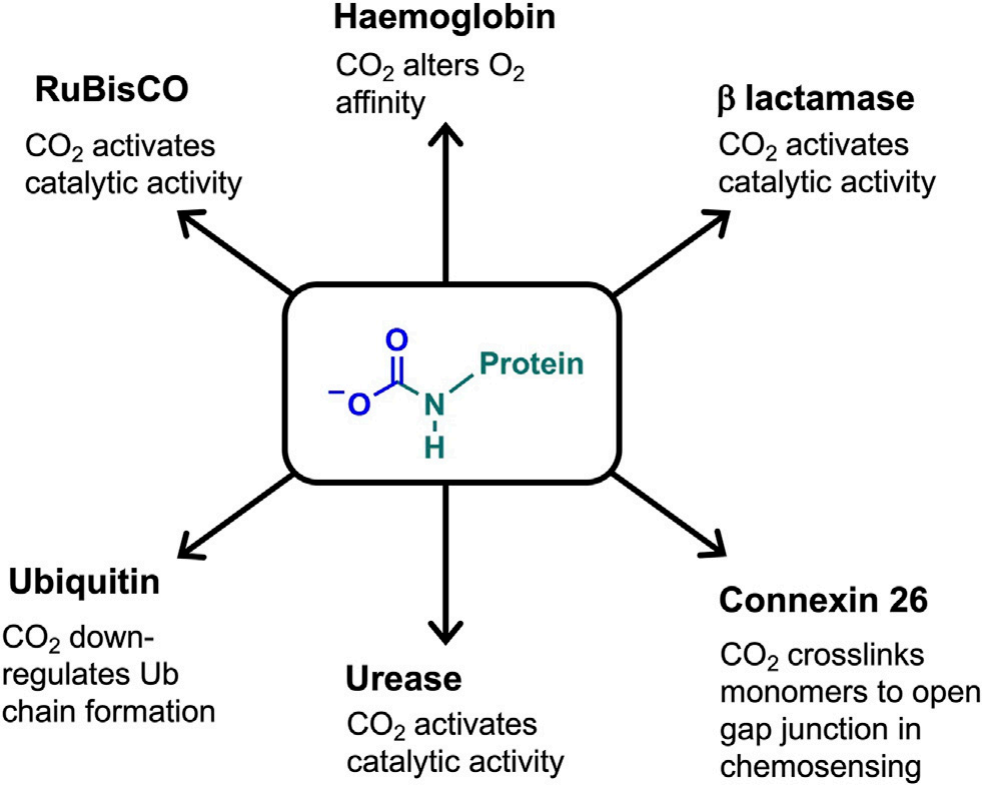
Defined role of the carbamate PTM in a selection of CO_2_-binding proteins.

Carbamates have been observed on specific lysine side chains in the crystal structures of several proteins, e.g. class D β-lactamase (Golemi et al., 2001), urease (Yamaguchi and Hausinger, 1997), alanine racemase (Morollo et al., 1999), and transcarboxylase 5 S (Hall et al., 2004). Lysine carbamylation can be critical to protein/enzyme activity. Examples include having a direct role in catalytic activity (class D β-lactamase, (Golemi et al., 2001), being a co-catalytic determinant, which bridges metal ions, (e.g. urease, (Yamaguchi and Hausinger, 1997), or in promoting (through hydrogen bonding) active site rearrangements for protein function (e.g. alanine racemase; (Morollo et al., 1999). Figure 3 summarises the carbamate functional role in haemoglobin, RuBisCO and other proteins discussed below.

### Class D β-lactamase

β-lactam antibiotics are essential in treating bacterial infection, inhibiting bacterial growth by acylating an active-site serine in essential penicillin-binding proteins and preventing the cross-linking of peptide chains to form peptidoglycan. β-Lactamases enable resistance to β-lactam antibiotics. These enzymes hydrolyse compounds containing a β-lactam ring and are classified based on sequence motifs and mechanism of hydrolysis (Class A-D). Class D OXA enzymes are the most diverse and least well-understood β-lactamases (Maveyraud et al., 2000; Tooke et al., 2019). The class is widely dispersed, found in gram-negative and gram-positive bacterial species and mobilised horizontally predominantly by plasmids and integrons (Bush, 2018; Tooke et al., 2019). In 2000, the first crystal structures of OXA-10 of *Pseudomonas aeruginosa* were published (Maveyraud et al., 2000; Strynadka et al., 2000). Maveyraud et al., 2000 discovered that OXA-10 is a dimeric beta-lactamase. However, the overall topology of OXA-10 class D β-lactamase is like class A, although amino acid sequence identity is low. Significant structural differences were found in the active site of OXA-10 compared to corresponding regions in class A (and C β-lactamases). In its native state, OXA-10 Lys-70 is carbamylated. It was suggested that this carbamylation offered a possible relationship between enzyme activation by CO_2_ and anion inhibition (Maveyraud et al., 2000). Golemi et al., 2001 used activity assays and fluorescence-based approaches to confirm that Lys-70 carbamylation is reversible and that OXA-10 β-lactamase depends on Lys-70 for enzyme acylation and deacylation steps in catalysis. This finding was supported by the inability of Lys-70 mutants to support deacylation. It was suggested that the hydrophobicity of the OXA-10 active site lowered the Lys-70 p*K*_a_ favouring carbamylation (Golemi et al., 2001; Sun, 2003).

### Urease

Urease plays an essential role in nitrogen metabolism in archaea, bacteria, fungi, plants and invertebrates (Dixon et al., 1975; Krajewska, 2009; Rutherford, 2014). It catalyses urea hydrolysis to form carbonic acid (H_2_CO_3_) and ammonia (NH_3_) and is important in human disease, plant metabolism, and agricultural ammonia emissions (Krajewska, 2009; Svane et al., 2020). In bacteria, ureases’ active site and primary structure are well studied in organisms such as *Proteus mirabilis*, *Helicobacter pylori*, and *Klebsiella aerogenes* (Jabri et al., 1995; Jabri and Karplus, 1996). The mechanism of urease activation is CO_2_ dependent *in vitro* (Pearson et al., 1998). This dependence has been explained by the active site containing two Ni^2+^ ions, which are bridged (stabilised) by the carboxyl group of a carbamylated lysine residue (Lys-217) that is essential for urease activation (Jabri and Karplus, 1996; I.-S. Park and Hausinger, 1995).

## Computational Approaches for the Identification of Carbamate Sites

To enhance protein carbamate discovery, Jimenez-Morales et al. (2014) developed a model to predict the uncarboxylated and carboxylated status of lysine residues in proteins. They initially used a training set of 251 proteins (identified by X-ray crystallography) which contained at least one protein subunit with a carboxylated lysine residue (KCX) to investigate the characteristics of the carbamate microenvironment when compared to uncarboxylated (LYS) sites. They observed that the crucial feature of the KCX microenvironment “*was the large numbers of packed atoms, water molecules and ions found in proximity to the KCX site chain*.” Additionally, all KCX sites were buried (inaccessible from the surface), with residues converging structurally at the KCX site dispersed along the protein’s primary sequence (so no sequence motif was associated with lysine carboxylation). The KCX residue was often in contact with positively charged ions with mono-nuclear or bi-nuclear interactions with divalent ions (Zn^2+^, Mg^2+^, Co^2+^, Fe^2+^, Ni^2+^, Mn^2+^). His and Asp residues were present in all analyses of KCX containing metal-binding sites. No more than one KCX site was observed on any single protein chain. The RuBisCO carbamylated lysine (used in the training set) is typical of a structure where the carbamate binds Mg^2+^ coordinated by Asp and Glue residues (Figure 2).

This information was used to develop a naïve Bayesian model (to predict potential KCX and LYS sites (Predictor of Lysine Carboxylation: PreLysCarb)). The 251-protein data set was used for training/testing (along with sub-sets with redundancy reduction implemented at 40 and 90% sequence identity). They carried out “leave-one-out cross-validation tests” on the three data sets. At 90% sequence identity PreLysCar correctly classified 54/62 KCX sites (87% sensitivity) and 4255/4259 LYS sites (99.7% specificity). Investigating false-positive rates in high-resolution protein structures, the model indicated that 11 to 19/575 proteins were incorrectly predicted to have a KCX residue (false positive rate of between 1.9 and 3.3%). When PreLysCar was applied to a subset of solved protein structures from the PDB (structures greater than 200 residues, solved by X-ray crystallography, containing 14,261 protein chains after 90% redundancy removal), it predicted that at least 1.3% of proteins with more than 200 amino acids in the PDB could potentially be subject to spontaneous lysine carboxylation. As the model has been trained using previously identified stable carbamates, which are predominantly buried, it may only represent a subset of possible KCX sites (Linthwaite et al., 2020).

What is clear from this analysis is that no consensus sequence enables easy CO_2_-binding site prediction as has proven so successful for phosphorylation sites, for example. This observation is borne out by analysing the growing numbers of experimentally observed carbamates (*see* following sections) with no clear primary consensus sequence. Future machine learning approaches as experimental data sets increase in size will, therefore, be essential as predictive tools.

## Mass Spectrometry Approaches for the Identification of Carbamate Sites

Mass spectrometry-based proteomics is a powerful tool for discovering and exploring PTMs. Although carbamylation of neutral *N*-terminal α-amino or lysine ε-amino groups may be a “*general and functionally important phenomenon throughout biology*” (Morrow et al., 1974) and “*form the basis for a widespread mechanism of biological regulation*” (Lorimer, 1983), the transient and readily reversible nature of this PTM all but renders impossible the use of mass spectrometry-based proteomics workflows (even when soft ionisation techniques are utilised; Terrier and Douglas, 2010). This ready reversibility limits our discovery of the distribution and contribution of carbamylation to biological functions.

Linthwaite et al., 2018 developed a chemical mechanism of covalently “trapping” carbamates under physiologically relevant conditions to overcome this limitation. In this technique, the triethyloxonium ion (TEO; a crystalline salt soluble under aqueous conditions) stabilises pre-formed carbamate modifications (formed through incubation of proteins or cellular lysates with CO_2_) by transferring an ethyl group from the oxonium ion to the negatively charged carbamate, forming a covalent bond which can withstand downstream protease digestion and enables CO_2_-binding protein identification by HPLC-ESI-MS/MS analysis. This approach has been validated for non-buried exchangeable carbamate binding sites (where presumably the carbamate is labile and the bound CO_2_ exchangeable into the bulk solvent) using individual amino acids and single proteins (previously known to be carbamylated; Linthwaite et al., 2018). This approach has recently contributed to studies of CO_2_ interaction with connexin 26 and ubiquitin (Sections 6.1 and 6.2) and shows potential in carbamate discovery through screening cell lysates (Section 6.3).

### CO_2_ and Connexin 26

Connexins (Cx) have an essential role in intercellular and extracellular communication and, therefore, homeostasis in multicellular organisms. These transmembrane proteins form hexameric connexons or hemichannels (HCs) in the plasma membrane, allowing low molecular weight molecules to transfer across the plasma membrane (Meigh et al., 2014). In closely apposed membranes, two HCs can dock together to form homomeric or heteromeric gap junctions (GJs), which mediate intercellular communication through the transfer of low molecular weight molecules (<1.1.5 kD), e.g. ions, metabolites and second messengers, between cells (Giepmans, 2004; Meigh et al., 2014; Willebrords et al., 2016). GJ intercellular communication can be regulated by pH (closed by acidification), transmembrane voltage (opened by voltage potential greater than −20 mV) and calcium concentration (opened by removal of extracellular Ca^2+^). PTMs such as S-nitrosylation, sumoylation and phosphorylation can directly regulate GJ opening (Willebrords et al., 2016).

Connexin 26 (Cx26) HCs and GJs are found in cells throughout the body, including in the cochlear epithelial network of the ear, keratinocytes of the skin, alveolar epithelium of the lungs, epithelial cells of the GI tract, and chemosensory areas of the brain (Cohen-Salmon et al., 2002; Huckstepp et al., 2010a; Huckstepp et al., 2010b; Meigh et al., 2014; Willebrords et al., 2016; Srinivas et al., 2018; van de Wiel et al., 2020). Mutations in the gene encoding Cx26 (GJB2) are relatively common (frequency of carriers ∼2–4% of the human population) and are linked to nine non-syndromic and syndromic deafness disorders (SDD; Cook et al., 2019; Srinivas et al., 2018). SDDs are associated with visual impairment and dermatological abnormalities, and in some instances, Keratitis-Ichthyosis-Deafness syndrome is underpinned by Cx26-A88V and Cx26-G45E missense mutations (Meigh et al., 2014).

Huckstepp et al., 2010b and Wenker et al., 2012 suggested that Cx26 has a role in mediating the central CO_2_-dependant drive to breathe with HCs enabling ATP release from the medulla oblongata in the absence of extracellular acidification. Exploring the potential molecular mechanism, Huckstepp et al., 2010a found that increasing pCO_2_ at fixed pH opens Cx26 HCs (and HCs of two related beta-connexins, Cx30 and Cx32). Meigh et al., 2013 compared the amino acid sequences of Cx26, Cx30 and Cx32 with Cx31 (a connexin that has no sensitivity to pCO_2_) and hypothesised a carbamylation motif present in Cx26, 30, 32 that was absent from Cx31. Using the existing Cx26 crystal structure (Maeda et al., 2009), the authors noted that the carbamylation “motif” contained K125 at the end of a subunit’s alpha helix where K125 is oriented towards R104 on a neighbouring subunit of the hexamer. Therefore, they hypothesised that if K125 was carbamylated, it could feasibly form a salt bridge (“carbamate bridge”) with R104, linking the subunits, and preventing hemichannel closure. By inserting the identified carbamylation “motif” into Cx31, they demonstrated that it was sufficient to form a CO_2_-sensitive hemichannel. If the K125 residue was substituted for an amino acid that could not be carbamylated, CO_2_ sensitivity was lost. Using Cx26, they confirmed that K125 and R104 were essential for forming the carbamate bridge. Meigh et al., 2015 used a similar mutation-based approach to explore the potential for alternative mechanisms of bridge formation between residues 125 and 104 in adjacent hexamer subunits. They found they could convert the CO_2_-sensitive hemichannels to a NO/NO^2−^ sensitive hemichannel using Cx26-K125C with Cx26-R104 or a redox-sensitive hemichannel using the combination of Cx26-K125C and Cx26-R104C, thus suggesting that distinct mechanisms of bridging involving residues 125 and 104 on adjacent hexamer subunits of Cx26 was possible (Meigh et al., 2015).

Using a combination of carbamate trapping, high-resolution cryo-EM and classification of particles, Brotherton et al., 2020 proposed that under physiologically relevant high pCO_2_ conditions (90 mmHg), a carbamate was formed on Lys-125, and additionally at two other positions Lys-108 and Lys-122, but not under physiologically relevant low pCO_2_ conditions (20 mmHg). They suggested that the shared environment within the cytoplasmic TM2 and TM3 regions of the Cx26 mobile loop favoured CO_2_ modification. Classification of particles indicated that the positions of TM2 and TM3 could influence the conformation of the *N*-terminal helix and found that under high pCO_2_ conditions, the *N*-terminus was more defined than under low pCO_2_ conditions. The authors hypothesised that gating is mediated by the movement of the *N*-terminal helix and its ability to plug the channel under physiologically relevant conditions (Brotherton et al., 2020).

Recent work by Nijjar et al., 2020 has shown in contrast to the opening of HCs at high physiological CO_2_ that intact Cx26 GJs connecting HeLa cells are closed when pCO_2_ is increased from 35 to 55 mmHg. This closing effect is dependent on the same residues (K125 and R104) involved in the CO_2_-dependant opening of Cx26 hemichannels. Further, the action is also directly attributed to a change in pCO_2_ rather than to changes in pH. They explained the contrasting action based on the free energy difference between CO_2_-bound and unbound states for HC and GJ (Nijjar et al., 2020). Specifically, it was energetically more favourable for HCs to bind CO_2_ in the open state and less energetically unfavourable to close. In contrast, it was energetically more favourable for the gap junction to bind CO_2_ in the closed state and then energetically unfavourable to open. The docking of two connexons as an HC provided a close interaction that constrained the conformation of hemichannels docked in a gap junction. Niijar et al., 2020 proposed the need for more information about the structures of Cx26 as free HCs and as components of GJs to investigate conformational differences and provide a greater understanding of the differential modulation of hemichannels and gap junctions by CO_2_. What is clear from these studies is that CO_2_ can have a signalling role *via* carbamate formation.

### CO_2_ and Ubiquitin

Ubiquitin (Ub) is a highly conserved protein found in all eukaryotic cells. It plays a crucial role in regulating protein activity and degradation through Ub protein covalent conjugation to a lysine side chain on target proteins (Komander, 2009). Ubiquitylation conjugation occurs through the sequential activity of Ub-activating enzymes (E1), Ub-conjugating enzymes (E2), and Ub ligases (E3) (Yau and Rape, 2016; Heap et al., 2017) and can be reversed by deubiquitylating enzymes (DUBs). Ub can be conjugated into poly-Ub chains through the *N*-terminus (M1) or at any of the seven conserved lysine residues (K6, K11, K27, K29, K33, K48, and K63) forming single, mixed or branched Ub-chains, resulting in a diverse range of Ub-chain structures (Swatek and Komander, 2016; Yau and Rape, 2016; Heap et al., 2017). Distinct Ub-chains are involved in different biological functions (Heap et al., 2017). The most abundant and well-studied of these are K48 single linked Ub-chains which target proteins for proteasomal degradation (Heap et al., 2017), and K63 single linked Ub-chains involved in cell signalling, trafficking and lysosomal degradation (Ohtake et al., 2015; Swatek and Komander, 2016; Michel et al., 2017). Roles of other less abundant single and mixed linked Ub-chains include regulation of enzymatic activity (K33), cell cycle regulation (K11), inflammation and immune response (M1, K63/M1, K48) and post-replication repair (K6 and K33) (Komander, 2009; Michel et al., 2017).

Ub activity can be further regulated by additional PTMs, e.g. acetylation, phosphorylation and SUMOylation (Koyano et al., 2014; Morimoto and Shirakawa, 2016; Lamoliatte et al., 2017; Lacoursiere et al., 2020). For example, all Ub lysine residues (except K29) are acetylated under differing cellular conditions (Choudhary et al., 2009; Elia et al., 2015; Lacoursiere et al., 2020). Ohtake et al., 2015 demonstrated that when endogenous Ub is acetylated at K6 and K48, this does not affect the ability of Ub to conjugate with substrate protein but inhibits the elongation of K11, K48 and K63-linked Ub-chains by several E2 enzymes (by neutralising the lysine residue positive charge involved in non-covalent interaction of Ub with specific E2s). This acetylation results in the accumulation of monoubiquitylated substrates in the cell. Using a SILAC based approach, Ohtake et al., 2015 confirmed that acetylation of K6 and K48 was linked to the enrichment of chromosome or chromatin related factors, including histone H2B, and that monoubiquitylated H2B was stabilised by the expression of acetylated Ub (Ohtake et al., 2015; Morimoto and Shirakawa, 2016; Michel et al., 2017). It has recently been suggested that Ub PTMs add an extra layer of complexity to the ‘ubiquitin code’ and that this extends beyond currently identified PTMs (Ohtake and Tsuchiya, 2016).

**FIGURE 4:**
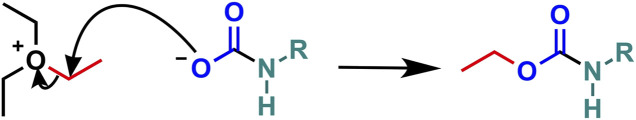
Trapping a protein carbamate with TEO. TEO transfers an ethyl group (red) to the anionic carbamate derived from CO_2_ (blue) and protein primary amine (green).

Linthwaite et al., 2021 recently explored the potential modification of human Ub by CO_2_. They demonstrated that Ub K33 and K48 could be carbamylated under physiological conditions by CO_2_ (25 mM CO_2_/HCO_3_ at pH 7.4), using the “trapping” method (Figure 4) outlined in Linthwaite et al., 2018. Ub carbamylation was confirmed independently by ^13^C-NMR, identifying K6 and K63 as additional carbamylation sites. Using *in vitro* conjugation assays, Linthwaite et al. (2021) found that di-Ub formation at K48 was significantly decreased when the CO_2_ concentration was increased from the normal physiological reference range (1.8–2.3 mM dissolved CO_2_) to the hypercapnic range (>2.3 mM CO_2_).

The effect of elevated CO_2_ on Ub-dependant processes was explored and focused on the regulation of nuclear factor κB (NF-κB) (Iwai, 2014). NF-κB is a transcription factor central to inflammation, innate and acquired immune responses, nervous system function, and cell survival (Iwai, 2014; Albensi, 2019; Song and Li, 2021). Under elevated pCO_2_, NF-kB mediated transcription is suppressed (Cummins et al., 2010). NF-κB is inactive in resting cells, bound in the cytoplasm to the inhibitor of NF-KBs (IκBs) proteins, blocking its transport to the nucleus. There are two known pathways of NF-κB activation, the canonical and non-canonical pathways. In the canonical pathway, the IκB kinase complex (IKK1, IKK2 and NF-κB essential modulator (NEM)) is activated upon interaction with, e.g., inflammatory cytokines or Toll-like receptor (TLR) ligands. This activation results in specific phosphorylation of IκB Ser residues within IκBs. Phosphorylated IκBs are recognised by the Ub ligase complex SCF^βTrCPs^ to generate K48-linked poly-Ub chains and are subsequently degraded by the proteasome. NF-κB is released from IκBs and translocates to the nucleus, where it binds to the DNA consensus sequence of a target gene. TNF-receptor family proteins activate the non-canonical pathway. This activation results in the stabilisation of NIK and its phosphorylation of IKKα. This phosphorylated IKKα induces further phosphorylation of NF-κB2/p100, which forms a complex with RelB. Once phosphorylated NF-κB/p100 is ubiquitinated by SCF^βTrCPs^ E3s generating K48-linked poly-Ub chains. This ubiquitinylation is followed by the partial degradation to p52 by the proteasome. The resulting RelB/p52 heterodimer is translocated to the nucleus (Iwai, 2014).

When cells carrying an NF-κB dependent GFP reporter were exposed to increasing TNFα concentrations under normal (5% (v/v) CO_2_ in air) and elevated CO_2_ conditions (10% (v/v) CO_2_ in air), NF-kB dependent GFP reporter activity was significantly decreased under elevated CO_2_ (Linthwaite et al., 2021). Transfection of the same cells with plasmids encoding wild-type Ub, mutant K48R Ub, mutant K63R Ub, or an empty vector was used to address the hypothesis that overexpression of K48R Ub would alter the relative response of the NF-kB pathway to elevated CO_2_. Cells transfected with an empty vector, Wt Ub or K63R Ub, showed an unaltered CO_2_ response. In contrast, when cells were transfected with K48R Ub, the effect of increased CO_2_ on the inhibition of the NF-κB reporter was ablated, suggesting that Ub K48 may be the target for CO_2_ in the NF-KB-dependent transcriptional response to hypercapnia in human cells.

### Mass Spectrometry-Based Approaches for Discovery of Carbamate Sites in the Proteome

The TEO-based carbamate trapping method combined with HPLC-MS/MS has also been used to discover carbamylated proteins in whole-cell lysates. A screen of an *Arabidopsis thaliana* cell lysate, which corresponded to 6% of the total proteome (3614 proteins/25,000 proteins), identified eight CO_2_-binding sites (i.e. lipid-transfer protein (Lys-K65), Rubisco Large Subunit (Lys-185), Peroxidase (Lys-262 and Lys-268), FBA1 (Lys-293), eukaryotic aspartyl protease family protein (Lys-251), PSBQA (Lys-109), and Fe Superoxide dismutase 1 (Lys-208) (Linthwaite et al., 2018)). Similarly, a screen of an *Escherichia coli* lysates corresponding to 14% of the total proteome (294/4300 proteins) identified six CO_2_ -binding sites (Linthwaite and Cann, 2021). In this instance, these included proteins involved in cellular processes identified as responsive to CO_2,_ i.e. assisting in the refolding of stress-denatured proteins, i.e. 60 kDa chaperone, carbamylated at Lys-34; (Kerner et al., 2005), preventing denaturation of DNA under extreme conditions (histone-like DNA HU-α, Lys-67; (Oberto et al., 2009), and proteins not previously identified in cellular processes responsive to CO_2,_ i.e. glutamine-binding periplasmic protein (Lys-127), ribose import binding protein RbsB (Lys-45 and Lys-285), and tryptophanase (Lys-121). In the future, this approach could be optimised for rapid screening of proteomes for non-buried exchangeable CO_2_-binding sites by including protein and/or peptide fractionation steps to increase proteome coverage and through the development of mechanisms of carbamate enrichment.

## Conclusion

We can draw some clear conclusions from the studies presented. First, carbamylation is a non-enzymatic PTM that can also be readily reversible. Second, the biological consequences of the carbamate PTM can vary discretely with changing CO_2_. Third, the carbamate PTM is more widespread among proteomes than suspected. Several future challenges arise from these conclusions. First, the extent of carbamylation in a proteome is unknown. More extensive proteomics analyses can address this challenge. However, such studies might benefit from developing enrichment methods and parallel computational approaches for carbamate prediction. Second, once the complement of carbamates in a proteome is known, how do we identify those that might have a functional role in CO_2_ sensing and signalling instead of forming through standard physicochemical mechanisms and being functionally neutral? Third, is carbamylation the sole mechanism for CO_2_ detection? If not, what other means exist, and how do we identify them? It is clear; there is plenty more to do in CO_2_ detection.
